# Supplementary material for: Macrophage Profiling in Head and Neck Cancer to Improve Patient Prognosis and Assessment of Cancer Cell–Macrophage Interactions Using Three-Dimensional Coculture Models
Source: Int J Mol Sci. 2023 Aug 15;24(16):12813. doi: 10.3390/ijms241612813 (PMC10454490; doi:10.3390/ijms241612813)
Supplement: Supplementary file 1 [file ijms-24-12813-s001.zip › ijms-2367588-supplementary.pdf]

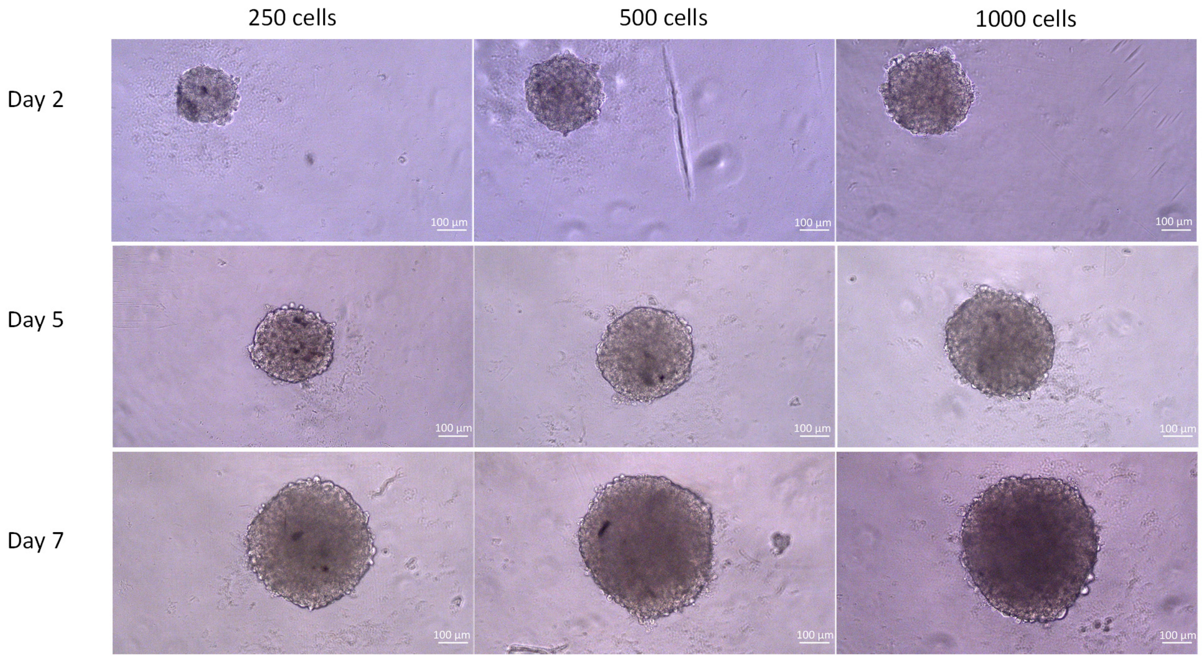

Figure S1: Visualization of spheroids growth according to the time and to the quantity of seeded cells.
